# Supplementary material for: Radiomics for the Prediction of Overall Survival in Patients with Bladder Cancer Prior to Radical Cystectomy
Source: Cancers (Basel). 2022 Sep 13;14(18):4449. doi: 10.3390/cancers14184449 (PMC9497387; doi:10.3390/cancers14184449)
Supplement: Supplementary file 1 [file cancers-14-04449-s001.zip › cancers-1900228-supplementary.pdf]

cancers

MDPI

Article

Radiomics for the Prediction of Overall Survival in Patients with Bladder Cancer Prior to Radical Cystectomy

Piotr Woźnicki [1], Fabian Christopher Laqua [1], Katharina Messmer [2], Wolfgang Gerhard Kunz [3], Christian Stief [2], Dominik Nörenberg [4], Andrea Schreier [5], Jan Wójcik [6], Johannes Ruebenthaler [3], Michael Ingrisch [3], Jens Ricke [3], Alexander Buchner [2], Gerald Bastian Schulz [2] and Eva Gresser [3,*]

[1] Department of Diagnostic and Interventional Radiology, University Hospital Würzburg, Würzburg-Oberdürrbacher Str. 6, 97080 Würzburg, Germany
[2] Department of Urology, University Hospital, LMU Munich, Munich-Marchioninistr. 15, 81377 Munich, Germany
[3] Department of Radiology, University Hospital, LMU Munich, Munich-Marchioninistr. 15, 81377 Munich, Germany
[4] Department of Radiology and Nuclear Medicine, University Medical Center Mannheim, Mannheim-Theodor-Kutzer-Ufer 1–3, 68167 Mannheim, Germany
[5] Department of Otolaryngology, University Hospital, LMU Munich, Munich-Marchioninistr. 15, 81377 Munich, Germany
[6] Faculty of Medicine, Medical University of Warsaw, Żwirki i Wigury 61, 02091 Warsaw, Poland
* Correspondence: eva.gresser@med.uni-muenchen.de; Tel.: +49-89-4400-73620

Table of contents:

1. **Supplementary Figure S1**. Comparative Kaplan-Meier analysis.
2. **Supplementary Table S1.** CT acquisition parameters and scanner models.
3. **Supplementary Table S2.** Parameters of the final Cox regression models.
4. **Supplementary Table S3.** All features selected for the models with their corresponding coefficients.
5. **Supplementary File S1.** Feature extraction parameters.

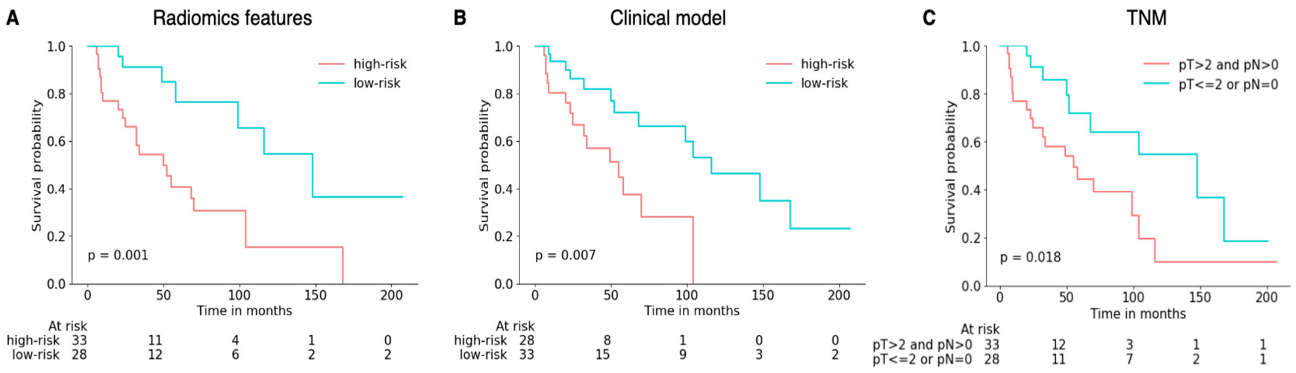

Supplementary Figure S1. Comparative Kaplan-Meier analysis for (**A**) the radiomics-based model, (**B**) the clinical model and (**C**) the model based on TNM classification. Predictions of each model were dichotomized into high- and low-risk groups according to the median risk score. The log-rank test was used to compare risk groups.

Cancers **2022**, *14*, 4449. https://doi.org/10.3390/cancers14184449 www.mdpi.com/journal/cancers

Supplementary Table S1. CT acquisition parameters and scanner models (median and min-max range in parentheses).

**Acquisition Parameters**	
Slice thickness [mm]	5 (0.75-6.0)
In-plane resolution	0.78 (0.55-1.07)
Tube voltage (kVp)	120 (90-140)
Tube current (mA)	158 (5-550)
Matrix	512 x 512
Kernel:	
I30f	78
B30f	72
B31f	40
B41s	19
other	92
**CT scanners**	
SIEMENS	270 CT scans<br>25 scanner models
TOSHIBA	17 CT scans<br>4 scanner models
GE	8 CT scans<br>5 scanner models
other	6 CT scans

Supplementary Table S2. Parameters of the final Cox regression models.

Model	# Features as Input	# Features with Non-Zero Coefficients*	L1 Ratio	Alpha
Clinical model	9	9	0.1	0.019
Radiomics features:				
intra (BCa + LN)	66	11	0.2	0.391
intra + peri (BCa)	66	4	0.9	0.087
intra + peri (BCa + LN)	132	8	0.9	0.087
Combined model	141	74	0.2	0.028

* Number of features selected by the penalized Cox model for prediction.

Supplementary Table S3. All features selected for the models with their corresponding coefficients.

**Combined Model**	
**Feature**	**Coefficient**
pT = 4	0.5573887580021498
R1+	0.5073042453051607
pN+	0.30865857222818377
firstorder_Median_intra_pt	-0.29260575739494654
pT >= 3	0.27536065388436715
age	0.26593759357430713
firstorder_Energy_peri_ln	0.24946152019506287

firstorder_Energy_intra_ln	-0.24618699147661807
shape_VoxelVolume_intra_pt	0.24552377509739146
firstorder_Variance_intra_ln	0.2443149534479168
shape_MeshVolume_intra_pt	0.2359968674576059
firstorder_Uniformity_peri_pt	-0.23331199721287174
firstorder_TotalEnergy_intra_pt	0.22496525473898943
shape_Maximum2DDiameterColumn_intra_pt	-0.21937942295548896
firstorder_10Percentile_peri_ln	0.2149650637035281
shape_Flatness_peri_ln	0.21419298614133775
firstorder_Entropy_intra_ln	-0.21146972801106315
shape_Maximum2DDiameterRow_intra_ln	0.19190043803427595
shape_Maximum2DDiameterColumn_peri_pt	-0.1788578347218928
firstorder_RobustMeanAbsoluteDeviation_peri_pt	-0.17756973144250415
shape_SurfaceVolumeRatio_intra_ln	-0.17423237058841468
firstorder_Variance_intra_pt	-0.16814134807176592
male_gender	-0.1629426738996482
shape_MajorAxisLength_intra_ln	0.15356340092752813
shape_SurfaceVolumeRatio_intra_pt	-0.13589454875244286
shape_Maximum2DDiameterSlice_intra_ln	-0.12966273480266485
firstorder_Energy_peri_pt	-0.12956018379219014
firstorder_Range_intra_pt	0.11601310075220576
shape_LeastAxisLength_intra_pt	-0.11600723500125985
firstorder_Skewness_intra_pt	-0.11461709631890231
firstorder_TotalEnergy_intra_ln	-0.1056890128215974
firstorder_Skewness_intra_ln	0.10382846879110674
firstorder_Maximum_intra_pt	0.10132542732000718
shape_Sphericity_peri_pt	-0.09376413493696441
firstorder_Kurtosis_intra_ln	-0.0823602430069151
shape_Elongation_intra_pt	0.08167422008979472
firstorder_Mean_peri_pt	0.07786307765668031
shape_Maximum2DDiameterSlice_intra_pt	0.07780018781682416
firstorder_RootMeanSquared_peri_pt	0.07636843592451187
shape_SurfaceArea_intra_pt	-0.07571467577052762
shape_Elongation_peri_pt	-0.06793182640182462
shape_MinorAxisLength_peri_ln	-0.06609436087330477

firstorder_Range_peri_ln	0.06500725762781205
shape_SurfaceArea_peri_pt	-0.06286857153038773
firstorder_Minimum_peri_pt	-0.061304146075164705
firstorder_Minimum_intra_pt	-0.05991555911050594
firstorder_Mean_peri_ln	0.05914658430655499
firstorder_Energy_intra_pt	0.05896334892675439
firstorder_Uniformity_peri_ln	-0.05441445567650102
firstorder_Minimum_intra_ln	0.049301794824387596
firstorder_RobustMeanAbsoluteDeviation_intra_ln	0.04802595548335101
firstorder_TotalEnergy_peri_ln	0.04625722460849467
firstorder_Maximum_peri_ln	0.04022437588238521
firstorder_InterquartileRange_intra_ln	0.03894423223758351
shape_MeshVolume_peri_pt	-0.035758251534618604
shape_VoxelVolume_peri_pt	-0.032018069669831395
firstorder_InterquartileRange_peri_ln	-0.031881353664308026
firstorder_90Percentile_intra_ln	0.030703779769660093
firstorder_Range_peri_pt	0.03010344635635724
shape_Maximum2DDiameterRow_intra_pt	0.025793216278818677
shape_SurfaceVolumeRatio_peri_ln	0.02150295380790737
firstorder_RootMeanSquared_peri_ln	0.016380565226094112
firstorder_InterquartileRange_peri_pt	-0.01604307019740029
shape_Flatness_intra_ln	0.015468867977010033
shape_Flatness_intra_pt	0.01184791054346095
firstorder_Median_peri_ln	0.011216991541617764
firstorder_Uniformity_intra_ln	0.010911122254329829
firstorder_Minimum_peri_ln	-0.006033013236992288
shape_Maximum2DDiameterColumn_intra_ln	-0.005784146506453603
firstorder_MeanAbsoluteDeviation_intra_ln	0.004082902791016376
pT >= 1	0.001144593453195357
pT >= 2	0.0010580833744271826
firstorder_Kurtosis_peri_ln	-0.000714622790730724
shape_MeshVolume_intra_ln	-0.00023892843482439994
**Clinical model**	
**Feature**	**Coefficient**
pT = 4	0.7010803727897716

R1+	0.5932421242575134
male_gender	-0.3280667049772867
pN+	0.3124377147266026
pT >= 3	0.30958297766867665
age	0.30182519767366234
pT >= 2	0.12474511942451086
pT >= 1	0.12458232517761718
lymphovascular_invasion	0.10209096725997804
**Radiomics model**	
**Feature**	**Coefficient**
firstorder_Range_peri_pt	0.1413816537429998
firstorder_10Percentile_peri_ln	0.13723303513809565
shape_SurfaceVolumeRatio_intra_pt	-0.08506570438822375
firstorder_Entropy_peri_pt	0.027769862393778443
shape_VoxelVolume_intra_pt	0.023221222609135523
shape_MeshVolume_intra_pt	0.0133937874121474
firstorder_Maximum_peri_ln	0.011488654061533356
shape_LeastAxisLength_peri_ln	0.006037228031560803
**Radiomics model (only intratumoral features)**	
**Feature**	**Coefficient**
shape_SurfaceVolumeRatio_intra_pt	-0.07584783909880814
shape_VoxelVolume_intra_pt	0.03380911156355748
shape_MeshVolume_intra_pt	0.03263411784620046
firstorder_TotalEnergy_intra_pt	0.028747666188233205
shape_LeastAxisLength_intra_ln	0.021262172132302273
firstorder_Minimum_intra_pt	-0.011731537754111943
shape_Maximum2DDiameterRow_intra_pt	0.005538865013385587
firstorder_90Percentile_intra_ln	0.005387050994644549
firstorder_Maximum_intra_ln	0.004265203402206109
firstorder_RootMeanSquared_intra_ln	0.003576705633835477
firstorder_Mean_intra_ln	0.0026283059638322853
**Radiomics model (only primary tumor)**	
**Feature**	**Coefficient**
firstorder_Range_peri_pt	0.16344211778945497
shape_SurfaceVolumeRatio_intra_pt	-0.10997460702413568

shape_VoxelVolume_intra_pt	0.027365494672709285
shape_MeshVolume_intra_pt	0.020412968276585754

Supplementary File S1. Feature extraction parameters.

imageType:
 Original: {}

featureClass:
 firstorder:
 shape:

setting:
 normalize: false
 normalizeScale: 500
 interpolator: 'sitkLinear'
 resampledPixelSpacing: [2, 2, 2]
 binWidth: 25
 voxelArrayShift: 1000
 label: 1
